# The effect of body fat mass and fat free mass on migraine headache

**Published:** 2013

**Authors:** Soodeh Razeghi Jahromi, Maryam Abolhasani, Alipasha Meysamie, Mansoureh Togha

**Affiliations:** 1 Assistant Professor, Geriatric Group, Sina Hospital AND Endocrine and Metabolic Research Center, Obesity Group, Tehran University of Medical Sciences, Tehran, Iran; 2 Assistant Professor, Sport Medicine Group, Sina Hospital AND Endocrine and Metabolic Research Center, Obesity Group, Tehran University of Medical Sciences, Tehran, Iran; 3 Associate Professor, Department of Community and Preventive Medicine, School of Medicine, Tehran University of Medical Sciences, Tehran, Iran; 4 Professor, Department of Neurology, Sina Hospital AND Iranian Center of Neurological Research, Tehran University of Medical Sciences, Tehran, Iran

**Keywords:** Migraine, Fat Free Mass, Truncal Fat Free Mass, Obesity

## Abstract

**Background:**

Obesity seems to be associated to migraine headache. Increase in body fat, especially in gluteofemoral region, elevates adiponectin and leptin secretion which in turn impair inflammatory processes that could be contributing to migraine risk. This study was designed to assess the relationship between body composition and risk of migraine for the first time.

**Methods:**

In this cross-sectional study, 1510 middle-aged women who were visited in a weight reduction clinic of university were recruited. Migraine was diagnosed with HIS criteria. Body composition parameters including total fat mass (FATM), total fat free mass (FFM), truncal fat mass (TFATM), and truncal fat free mass (TFFM) was assessed using bioelectric impedance. We further assessed cardiovascular risk factors and smoking as confounding factors. To determine the real association between different variables and risk of migraine, the associations were adjusted by multivariate logistic regression analysis.

**Results:**

Elevation in fasting blood sugar, total cholesterol, LDL cholesterol, FFM, TFFM, and waist-to-hip ratio increased the risk of migraine. When the associations were adjusted for other factors, only the association between migraine and FFM remained statistically significant.

**Conclusion:**

Lower FFM increased the risk of migraine in overweight and obese individuals. In the other words, higher fat free mass could be a protective factor for migraine.

## Introduction

Obesity is one of the major public health problems. The prevalence of obesity is increasing consistently worldwide.^1^ In Iran, the prevalence of overweight and obesity were 57% in women and 42.8% in men.^2^ Overweight and obese individuals are at increased risk for a variety of disease including metabolic syndrome, diabetes mellitus, arthritis, gout, cardiovascular disease, and cancer.

The association between migraine and obesity has also been reported.^3, 4^ Studies also suggest a relationship between obesity and migraine frequency and severity as well as migraine features such as phonophobia and photophobia.^1^ Subcutaneous fat, especially in the gluteofemoral region in women, seems to increase leptin and adiponectin secretion. Leptin and adiponectin elevation in turn impairs insulin sensitivity and mediates inflammatory process attributed to migraine risk.^5^ The evidences about the relationship between body composition (fat mass/fat free mass) and migraine are rare. Current study was designed to assess the relationship between migraine and body composition in overweight and obese individuals.

## Materials and Methods

### Study population

This was a cross-sectional study among overweight and obese healthy women who were visited for weight reduction in Obesity Clinic of Sina University Hospital, Tehran, Iran, between February 2009 and March 2013. 1630 women were eligible to participate in this study. From these subjects, 1510 overweight and obese women with mean age of 38.5 ± 12.6 years accepted to participate. All women signed written informed consent. Thus a total of 1510 women with BMI ≥ 25 and history of the migraine headache were included in the study. 25 women with missing data of BMI or migraine headache were excluded.

### Assessment of migraine

Migraine was diagnosed in accordance with the International Headache Society (IHS) criteria. Patients with headache were classified as migraineurs if they fulfilled the following criteria: (1) Headache attacks lasting 4–72 hours (< 4 hours was accepted for those who reported often visual disturbances before headache); (2) headache with at least one of the following characteristics: Pulsating quality, unilateral location or aggravation by physical activity; (3) presence of at least one of the following symptoms during headache: Nausea, photophobia, or phonophobia.

### Anthropometric measurements

Body weight was measured to the nearest 0.1 kg using Seca 755 Dial Column Medical Scale. Height was measured to the nearest 0.1 cm using a standard stadiometer. Body mass index (BMI) was calculated by dividing weight in kilograms by height in square meters. BMI ≥ 25 was defined as overweight and BMI ≥ 30 was defined as obesity. Waist circumference was measured by the standard tape meter at the maximal narrowing of the waist from anterior view. Hip circumference was measured at the point of maximal gluteal protuberance from the lateral view. Waist-to-hip ratio was calculated through dividing the waist circumference by hip circumference.

Body composition was measured by body composition analyzer type BC-418 MA TANITA. The participants were asked not to eat or drink within 4 hours, and not to exercise within 12 hours of the test. Participants were asked to void within 30 minutes left to the test (with empty bladder) and had minimal consumption of diuretic agents.

The body composition analyzer measured total fat mass (FATM), total fat free mass (FFM), total body water, and segmental analysis for fat mass and fat free mass in trunk.

### Cardiovascular risk factor assessment

Fasting blood sample was obtained to measure fasting blood sugar (FBS), total cholesterol, low density lipoprotein (LDL) and high density lipoprotein (HDL).

Hyperglycemia was defined as having FBS ≥ 126 mg/dl. The patient was diagnosed with dyslipidemia if he/she had serum levels of total cholesterol ≥ 240 mg/dL or LDL ≥ 160 mg/dL or HDL ≤ 40 mg/dL or was taking lipid lowering drugs.

Blood pressure was measured for participant in a sitting position after a 10-minute rest, on the upper arm, using mercury sphygmomanometer (Richter™). Hypertension (HTN) was defined as having systolic blood pressure ≥ 140 mmHg or diastolic blood pressure ≥ 90 mmHg. Smoking status was assessed through self-report. Smoking in past 6 months was defined as active smoking.

### Statistical analysis

For descriptive analysis of quantitative data, the mean ± standard deviation was used. For qualitative data, frequency percentage was reported. To evaluate the association between migraine and risk factors odds ratio (OR) with 95% confidence interval (CI) was used. Finally, we used multivariable logistic regression analysis to control the effect of confounding factors.

To determine the cut-off value of FATM, FFM, truncal fat mass (TFATM), and fat free mass (TFFM), the receiver operating characteristic (ROC) curve analysis was used with respect to BMI categories (BMI ≥ 30 kg/m^2^). Cut-off points were considered as the number which represented the maximum of the square of specificity plus sensitivity. Statistical Package for the Social Sciences, version 17.0 (SPSS, Chicago, IL, USA) was used to analyze the data.

## Results

**Figure 1:**
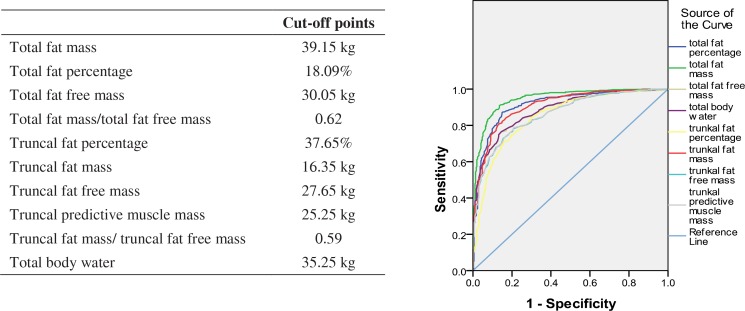
Receiver operating characteristic (ROC) curve of body composition parameters, according to body mass index categories BMI ≥ 30 was used as cut-off point. Cut-off points were considered as the number which represented the maximum amount of the sum of the square of sensitivity and specificity

Of the 1510 participants, a total of 320 (21.2%) were diagnosed with migraine. The participant's characteristics were summarized in Table 1. The independent variables were then categorized. For categorizing FATM, FFM, TFATM, and TFFM, cut-offs by ROC curves were applied (Figure 1).

**Table 1:** Characteristics of overweight and obese women participated in the study, categorized by having or not having migraine headache

	Migraineurs	Non migraineurs

	Mean ± SD	Mean ± SD
Age (year)	38.4 ± 11.1	38.5 ± 12.9
Weight (Kg)	88.9 ± 19.6	85.5 ± 19.1
Body mass index (Kg/m^2^)	35.5 ± 8.3	34.2 ± 6.9
Waist-to-hip ratio	0.86 ± 0.8	0.88 ± 0.8
Total fat mass (Kg)	38.5 ± 13.1	36.2 ± 13.4
Total fat percentage	42.1 ± 6.2	42.4 ± 13.4
Total fat free mass (Kg)	50.5 ± 8.1	50.9 ± 6.9
Truncal fat percentage	38.2 ± 6.0	36.7 ± 8.1
Truncal fat mass (Kg)	17.8 ± 5.4	16.9 ± 6.1
Truncal fat free mass (Kg)	27.8 ± 3.8	27.5 ± 3.5
Truncal predictive muscle mass (Kg)	26.6 ± 3.7	26.3 ± 3.2
Truncal fat mass/truncal fat free mass	0.6 ± 0.2	0.6 ± 0.2
Total body water (Kg)	37.1 ± 5.7	36.6 ± 5.0
Total cholesterol (mg/dL)	202.6 ± 39.2	192.9 ± 44.3
LDL-cholesterol (mg/dL)	121.6 ± 31.4	115.5 ± 36.3
HDL-cholesterol (mg/dL)	48.7 ± 11.4	47.3 ± 12.1
Fasting blood sugar (mg/dL)	96.6 ± 20.2	102.8 ± 30.9

The comparison of participants’ characteristics between migraineurs and non-migraineurs were presented in Table 2. Higher ratio of fat mass/fat free mass, truncal fat mass/truncal fat free mass, and volume of truncal fat mass were significantly associated with elevated risk of migraine. Except for FFM, the association between all the variables and migraine were attenuated after logistic regression analysis (Table 3).

**Table 2:** Association between migraine and biochemical, anthropometric, and other measured factors

	With migraine		Without migraine			

	No./total No.	Percent	No./total No.	Percent	Odds ratio (95% CI)	P
Total cholesterol ≥ 126 mg/dL	16/120	61.9	63/473	46.5	1.00 (0.56-1.81)	0.997
HDL-cholesterol ≤ 40 mg/dL	21/102	20.6	110/420	20.2	0.73 (0.43-1.24)	0.242
LDL-cholesterol ≥ 160 mg/dL	11/102	10.8	42/413	10.2	1.07 (0.53-2.16)	0.855
FBS ≥ 126 mg/dL	22/133	16.5	96/490	19.6	0.81 (0.49-1.35)	0.426
Waist-to-hip ratio < 0.88	153/268	57.1	542/961	57.1	1.03 (0.78-1.35)	0.840
BMI ≥ 30 kg/m^2^	203/278	74.1	674/978	68.9	1.22 (0.91-1.64)	0.188
FATM ≥ 39.15 kg	101/242	41.7	268/754	35.5	1.30 (0.19-1.75)	0.083
FFM ≤ 30.5 kg	242/243	99.5	749/754	99.3	1.62 (0.19-13.9)	0.659
FATM/FFM > 0.62	184/242	76.0%	241/754	32.0	1.49 (1.07-2.08)	0.018
TFATM ≥ 16.35	151/244	46.3	348/749	46.4	1.87 (1.39-2.51)	< 0.001
TFFM ≤ 27.65 kg	113/244	46.3	348/749	46.4	0.99 (0.74-1.33)	0.967
TFATM/TFFM > 59	154/244	63.11%	338/749	45.1	1.41 (1.05-1.89)	0.024
HTN	221/274	80.4	831/975	85.3	0.72 (0.51-1.02)	0.067
Smoking	8/269	2.97	17/970	1.7	1.72 (0.73-4.03)	0.208

FBS: Fasting blood sugar, BMI: Body mass index, FATM: Fat mass, FFM: Fat free mass, TFATM: Truncal fat mass, TFFM: Truncal fat free mass, HTN: hypertension

**Table 3:** Association of migraine and biochemical, anthropometric, and other measured factors in multivariate logistic regression analysis

		B	P	Odds ratio	95% CI	

					Lower	Upper
Step 1	Total cholesterol	0.045	0.921	1.04	0.43	2.54
	LDL-cholesterol	-0.252	0.610	0.77	0.29	2.05
	FBS	0.347	0.288	1.41	0.75	2.68
	Waist-to-hip ratio	-0.195	0.441	0.82	0.50	1.35
	FFM	0.570	0.127	1.77	0.85	3.68
	TFFM	0.175	0.474	1.19	0.74	1.92
Step 2	LDL-cholesterol	-0.222	0.570	0.80	0.37	1.72
	FBS	0.350	0.282	1.42	0.75	2.68
	Waist-to-hip ratio	-0.194	0.443	0.82	0.50	1.35
	FFM	0.577	0.116	1.78	0.87	3.65
	TFFM	0.174	0.475	1.19	0.74	1.92
Step 3	FBS	0.349	0.282	1.42	0.75	2.68
	Waist-to-hip ratio	-0.200	0.427	0.82	0.50	1.34
	FFM	0.463	0.132	1.59	0.87	2.90
	TFFM	0.171	0.482	1.19	0.74	1.91
Step 4	FBS	0.366	0.258	1.44	0.76	2.72
	Waist-to-hip ratio	-0.179	0.475	0.84	0.51	1.37
	FFM	0.562	0.039	1.75	1.03	2.99
Step 5	FBS	0.347	0.282	1.41	0.75	2.66
	FFM	0.429	0.030	1.53	1.04	2.26
Step 6	FFM	0.634	< 0.001	1.88	1.67	2.12

FBS: Fasting blood sugar, BMI: Body mass index, FATM: Fat mass, FFM: Fat free mass, TFATM: Truncal fat mass, TFFM: Truncal fat free mass, HTN: hypertension

## Discussion

In healthy overweight and obese individuals, elevation of total cholesterol, LDL-cholesterol, FBS, waist-to-hip ratio, FFM, and TFFM increased the risk of migraine. After adjustment, the only significant association was between migraine and FFM. At the tissue level, FFM focuses on skeletal muscle, bone, blood, and visceral organ. The main component is skeletal muscle.^6^

To the best of our knowledge, this is the first time that the relationship between body composition parameters and migraine was assessed. An energy-restricted diet in combination with aerobic and anaerobic exercises would reduce fat mass while preserving fat free mass.^7^ Therefore, restricting calorie intake and having an exercise program can reduce the risk of migraine in overweight an obese women.

Previously, large population based studies assessed the association between physical activity level and migraine. In line with our hypothesis about the effect of exercise on the risk of migraine, Milde-busch et al. in a study on 1260 adolescents concluded that there is a 3.4 fold risk of migraine in physically inactive individuals. They included migraine and tension-type headache, using the criteria of International Classification of Headache Disorders, 2^nd^ edition (ICHD-II).^8^ In the HUNT study, among a subset of 5847 adolescents, physical inactive, smoker, and overweight individuals were 1.4 fold more likely to suffer from recurrent headache (tension-type or migraine). Subjects with low physical activity level had 1.2 times greater risk for recurrent headache. In another large cross-sectional study of 46648 individuals, inactivity was associated with higher incidence of self-reported non-migraine and migraine headache.^9^

Moreover, some studies have assessed the therapeutic effects of aerobic exercise programs in migraineurs. Varkey et al. study has focused on increasing oxygen uptake and it was well tolerated.^10^ Evidences suggest that aerobic exercise increase beta endorphin secretion which may subsequently increase pain threshold.

Exercise is also associated with an increase in fat free mass. According to our findings, lower level of fat free mass was associated with increased risk of migraine. Therefore, physical activity can also reduce migraine risk by increasing fat free mass.

A number of limitations should be considered in interpretation of our results. First of all, our study population was limited to overweight and obese middle-aged women. Therefore, the results may not be expanded to other age-groups, men, or normal weight individuals. We chose overweight and obese women because they are more prone to migraine than men and normal weight subjects. Second, since this study was cross-sectional, although we adjusted the odd ratios for the most potential confounders, we cannot exclude the other possible confounding factors. Third, we just evaluated the association between body composition parameters and migraine. The association between body composition and other types of headache is remained to be evaluated.

## Conclusion

All together, the findings of the current study suggested that the decrease in fat free mass may increase the risk of migraine in overweight and obese individuals. Further studies are warranted to evaluate the effects of fat free mass changes on migraine severity.
